# Chronic cerebrospinal venous insufficiency: the end of “The Big Idea”?

**DOI:** 10.1002/brb3.308

**Published:** 2015-01-07

**Authors:** Erwin Stolz

**Affiliations:** Department of Neurology, Caritasklinikum SaarbrueckenRheinstrasse 2, D-66119, Saarbruecken, Germany

As early as 1863 Eduard Rindfleisch described and systematically studied the topographic relationship between multiple sclerosis (MS) plaques and brain tissue venules frequently found in the center of MS lesions (Rindfleisch 1863). In the 1930-ies and 40-ies Tracy Jackson Putnam took an interest in the relationship between MS plaques and the venous vasculature (Putnam 1933; Putnam and Adler 1937). By this time formation of venous thrombi in central venules of acute MS lesions had already been recognized. Putnam – as opposed to the fraction of researchers who explained these thrombi in plaque venules as an effect of a local “allergic reaction”—assumed an increased liability of MS patients to form cerebral venous thrombi, which he believed to be the cause of the inflammatory process. Based on this hypothesis he undertook a clinical trial on the effects of anticoagulation on the clinical course of MS with no convincing results (Putnam et al. 1947).

For the next 40 years it became silent around the vascular hypothesis in MS until in 1986 Schelling proposed a damaging effect of venous reflux to the brain and spinal cord as a pathogenetic aspect in MS (Schelling 1986). While Schelling assumed a mere mechanical effect of venous engorgement and reflux on the brain tissue, in 2006 Zamboni proposed an iron-dependent inflammation in multiple sclerosis induced by disruption of the blood-brain barrier on the venous side due to venous hypertension caused by disturbed cranial venous outflow and leading to iron deposition in brain tissue responsible for the ignition spark for the local inflammation (Zamboni 2006). He termed this hypothesis “The Big Idea.” In order to demonstrate disturbed cranial venous outflow in MS patients, Zamboni and his group developed five ultrasound criteria, four related to extracranial venous obstruction, one related to reflux into the deep cerebral veins. The syndrome of “chronic cerebrospinal venous insufficiency” (CCSVI) was supposed to be present when at least two of the five ultrasound criteria were diagnosed. In 2009 Zamboni and his group published landmark papers in which they reported a perfect separation of MS patients and controls based on CCSVI positivity resulting in a hitherto unheard of 100% sensitivity and 100% specificity in a clinical diagnostic setting (Zamboni et al. 2009a,b). Shortly afterward, astounding results of the effect of venous stenting on the clinical outcome in an open unblinded case series were published (Zamboni et al. 2009c). The term “Liberation Treatment” for this endovascular procedure was coined by Zamboni and his group.

While the scientific community remained cautious, “CCSVI” and “Liberation Treatment” gained tremendous public interest by promotion in print and film media and especially the new social media. There is a simple explanation to that: the concept of CCSVI is easy to grasp for nonprofessionals and treating CCSVI by angioplasty is logically consistent. CCSVI as a concept was indeed promoted as “The Big Idea.” Further, the term “Liberation Treatment” was extremely well chosen to attract attention of a wider audience. Especially in Italy and Canada health professionals and politicians were pressurized by patient support groups to offer endovascular treatment covered by the health insurance system despite the lack of controlled trials at that time.

In this issue of Brain and Behavior Tsivgoulis and coworkers published a systematic review on CCSVI and endovascular treatment in MS. There are three important messages: (1) if properly blinded, there is no difference between patients and healthy controls regarding CCSVI positivity, (2) in a large part studies reporting such a difference originate from Zamboni and his group, researches related to Zamboni and his group, or offering liberation treatment, and (3) in a sham-controlled, randomized, double-blind study design liberation treatment does not have an effect on clinical parameters. The authors are quite clear in their conclusion, that in view of several well conducted case–control studies contradicting the CCSVI concept in MS this hypothesis should be discarded. They regard the sonographic syndrome of CCSVI as poorly reproducible and clinically irrelevant. Further, Tsivgoulis and coauthors conclude that liberation treatment in MS may even be harmful regarding the clinical course.

As one of the CoSMo (CCSVI: studio Osservazionale Sclerosi Multipla e OND) study investigators I agree with their conclusions (Comi et al. 2013). However, was it really ethically justified to conduct endovascular trials in a situation when already the underlying hypothesis has come under fire? Now finally we have a class I sham-controlled, randomized, double blinded study on venous endovascular intervention in MS showing that there is no clinical benefit of the procedure (Siddiqui et al. 2014).

But is this really the end of the CCSVI concept and liberation treatment in MS? Both topics have been discussed too deeply in the social media and the blogosphere to suddenly disappear from the agenda. But the medical profession cannot act the innocent. Liberation treatment has become a profitable market. As long as all-round carefree packages are offered all around the world including toll-free numbers from the USA, Canada, and Europe, patient tourism to receive liberation treatment will not stop in the near future despite scientific lack of effectivity.
